# SO_2_ and HCHO over the major cities of Kazakhstan from 2005 to 2016: influence of political, economic and industrial changes

**DOI:** 10.1038/s41598-020-69344-w

**Published:** 2020-07-28

**Authors:** Zhuldyz Darynova, Mehdi Amouei Torkmahalleh, Talgat Abdrakhmanov, Serik Sabyrzhan, Sultan Sagynov, Philip K. Hopke, Jonilda Kushta

**Affiliations:** 1 Chemical and Aerosol Research Team, Department of Chemical and Materials Engineering, School of Engineering and Digital Sciences, Nazarbayev University, 010000 Nur-Sultan, Kazakhstan; 2 The Environment and Resource Efficiency Cluster, Nazarbayev University, 010000 Nur-Sultan, Kazakhstan; 3 Department of Public Health Sciences, University of Rochester School of Medicine and Dentistry, Rochester, NY 14642 USA; 4 Center for Air Resources Engineering and Science, Clarkson University, Potsdam, NY 13699 USA; 5 Energy Environment and Water Research Center, The Cyprus Institute, 2121 Nicosia, Cyprus

**Keywords:** Atmospheric science, Atmospheric chemistry

## Abstract

Satellite observations of the Ozone Monitoring Instrument (OMI) for tropospheric sulfur dioxide (SO_2_) and formaldehyde (HCHO) column mass densities (CMD) are analyzed for the period 2005–2016 over the atmosphere of Kazakhstan. Regarding SO_2_ the major hot spots relate to regions with high population and large industrial facilities. Such an example is the city of Ekibastuz that hosts the biggest thermal power plants in the country and exhibits the higher SO_2_ CMD at national level. The annual average CMD in Ekibastuz reaches 2.5 × 10^−5^ kg/m^2^, whereas for the rest of the country respective values are 6 times lower. Other hotspots, mostly urban conglomerates such as Almaty and Nur-Sultan, experience high CMDs of SO_2_ in particular years, such as 2008. One of the main reasons for this behavior is the financial crisis of 2008, forcing the application of alternate heating sources based on cheap low-quality coal. Regarding HCHO, an oxygenated Volatile Organic Compound (VOC), the main hot spot is noticed over the city Atyrau, the oil capital of the country where two massive oil fields are located. The highest HCHO CMD (9 × 10^15^ molecules/cm^2^) appears in the summertime due to secondary production as a result of the photo-oxidation of VOCs emitted by industrial sectors, oil refinery plants and vehicles. Strongly elevated HCHO amounts are also observed in Nur-Sultan in 2012 that could be due to the residential coal combustion and vehicle exhaust under poor winter dispersion conditions. Significant reductions in HCHO observed between 2012 and 2015 can be attributed to two significant measures implemented in the country in 2013 that aimed at the improvement of air quality: the introduction of the emission trading system (ETS) for greenhouse gases and Euro-4 standards for new vehicles entering the national vehicle fleet.

## Introduction

Sulfur dioxide (SO_2_) is emitted to the atmosphere from fossil fuel burning, mainly coal and residual oil as well as from volcanic eruptions. The atmospheric residence lifetime of sulfur dioxide is 2–7 days, and its main loss mechanism in the atmosphere is oxidation to form sulfuric acid (H_2_SO_4_). Atmospheric formaldehyde (HCHO) is mainly formed through the oxidation of different volatile organic compounds (VOCs) present in the atmosphere^1^. Apart from the photochemical oxidation, which contributes to 70–90% of the vertical column density (CMD) of atmospheric HCHO in urban areas^2^^,^ combustion, biogenic activities and biomass burning account for the majority of primary emissions. The loss of HCHO occurs by photolysis and reaction with hydroxyl radical (OH)^1^. HCHO levels play a significant role in controlling the ozone concentrations in urban areas since formaldehyde photolysis is an important source of hydroperoxy radicals^1^. The hydroperoxy radicals contribute to the ozone production cycle.

Long term monitoring of the atmospheric formaldehyde and sulfur dioxide by satellites may assist in determining the major sources of these compounds. Formaldehyde emissions have declined in the last 10 years in several cities including the USA (New York, Miami, and Philadelphia), Japan (Tokyo), and in several European cities (Paris, London)^3^. These reductions have potentially resulted from the cumulative effect of several emissions control policies regarding thermal power plants, emissions from vehicles, and the elimination of technically obsolete enterprises^4^. SO_2_ emissions also were reduced globally by 31% from 1990 to 2015^5^. However, individual regions contributed differently. For instance, during the first decade, large reductions in SO_2_ emissions happened in Europe and North America, 54% and 21% respectively. While in East Asia, emissions increased by 32% over this period. In the next decade, the situation remained similar for Europe and North America with emissions decreasing by 40% and 50%, respectively^6^. For East Asia, two different trends occurred: in 2000–2005, emission increased by 70%, and in 2005–2015, there were reductions of 13%^7,8^. In India during the whole 25 years, SO_2_ emission increased by 3.3 times and for Pakistan SO_2_ column density increased by 70% from 2004 to 2011^5,9^.

Economic indicators and energy data show that the gross domestic product (GDP) of Kazakhstan has multiplied by roughly a factor of six between 2002 and 2016^10^. Consequently, CO_2_ emissions increased by a factor of 1.5 for the period between 2005 and 2014^11^. Both the economic growth and the rise of CO_2_ emissions in Kazakhstan are related to oil and coal production as half of the energy of the country is produced from coal while the oil sector is the main source of economic income^10^. In 2014, Kazakhstan had the world’s highest household coal consumption per capita (157 kgcap) despite the fact that the country has an abundance of natural gas^12^. Simultaneously, the total level of non-methane volatile organic compounds (NMVOC) emissions increased from 41,300 tons/year in 2005 to 100,000 tons/year in 2016 from stationary anthropogenic sources^13^.

Sulfur dioxide CMDs were also driven mainly by anthropogenic sources such as energy production and industrial sectors, especially due to the high sulfur content of fossil fuels used in combustion processes and petroleum industries^7^. The dependence of the energy sector of Kazakhstan on raw material production highlights the importance of continuous monitoring of formaldehyde and sulfur dioxide to investigate their local and regional climate impacts. Ground monitoring of the SO_2_ and HCHO in Kazakhstan is limited due to small number of monitoring sites that employ outdated measurement methods^14^. Therefore, satellite retrievals provide a continuous approach to investigate the concentrations and trends of formaldehyde and sulfur dioxide levels in major cities of Kazakhstan.

Previous work regarding air pollution concentrations in Kazakhstan was based on stationary monitoring stations with limited measurements of pollutants in terms of species, duration, and spatial coverage. Only a few studies explored these compounds in detail^15–17^. Thus, the investigation of formaldehyde and sulfur dioxide using satellite retrievals in Kazakhstan is an important study.

The satellite monitoring method used in this work provides an independent and objective source of information, allowing conclusions to be drawn about changes in atmospheric composition. It gives opportunity to the decision makers to make policy decisions based on the pollution sources and their contribution to the local air quality. Such information regarding Kazakhstan and similar regions in the Central Asia is scarce in the literature.

This study investigated the temporal and spatial trends of HCHO and SO_2_ from 2005 to 2016 in the major cities of Kazakhstan with the highest population density, such as Nur-Sultan, Atyrau, Almaty, and Shymkent. HCHO concentrations were estimated using data from the ozone monitoring instrument (OMI). SO_2_ values were estimated using data from the Modern Era-Retrospective Analysis for Research and applications version 2 (MERRA-2) model.

## Materials and methods

The formaldehyde CMD values were retrieved from the Tropospheric Emission Monitoring Internet Service (TEMIS) that computes and distributes concentrations of global tropospheric and stratospheric trace gases and aerosols. TEMIS is a web-based service and is part of the European Space Agency that uses nadir-viewing satellite instruments such as GOME, OMI, SCIAMACHY, and ATSR^18^. The retrieval of sulfur dioxide CMDs was performed through the Giovanni website of the Physical Oceanography Distributed Active Archive Center (PO.DAAC) that collects data considering visual exploration and comparative analysis of physical oceanographic and climate information, including ocean winds, temperature, topography, salinity, and circulation^19^. In our study, data from the OMI instrument was used to estimate the formaldehyde and sulfur dioxide annual and seasonal concentrations in Kazakhstan because of its daily global coverage, small footprint, and its high spatial resolution that enables the detection of air pollution at urban scales.

The retrieval procedure for formaldehyde consists of three main steps. Initially, by the use of DOAS algorithm, the effective slant columns are obtained through the measured Earth reflectance spectra^20^. Secondly, the effective slant columns are converted to vertical columns by radiative transfer calculation of related air mass factors (including the presence of clouds and aerosols). The radiative transfer calculation of air mass factors to obtain vertical columns are performed by the method of Palmer et al.^21^. The third step includes a normalization of columns to eliminate remaining unphysical dependencies^22^. Our study uses a global monthly mean of the level-3 data for formaldehyde gas CMD retrieval.

The data retrieval for sulfur dioxide is performed in graph-based representation and values of column mass density (kg/m^2^) (CMD) were extracted to plot seasonal variations and trends. The SO_2_ retrieval process follows a similar procedure to formaldehyde gas. The SO_2_ slant columns are retrieved by OMI based on an algorithm of DOAS, with the wavelength range for sulfur dioxide being in 315–327 nm. The retrieval process consists of two steps. The first step is the retrieval of total ozone amount by comparing the radiances between calculation and actual measurement (317 and 331 nm). The second step considers the calculation of the difference between the determined and computed radiances involving four short UV wavelengths in the SO_2_ band: 310.8, 311.9, 313.2 and 314.4 nm^23^. In our study, the monthly average SO_2_ concentrations were used. The focus of our study were several urban conglomerates such as Almaty, Nur-Sultan, Shymkent and the industrial cities of Atyrau and Ekibastuz. More information about the trends in population density in these cities (for the period 2000 to 2018) and their location can be found in Annex I of the Supplementary Material.

### Time series decomposition

Time series data usually contain different types of patterns, and in order to capture the underlying patterns of our time series data, we first need to decompose it into several components, each of which represents a different structure in the data. *Time series decomposition* is a statistical task in which we deconstruct the data into several parts. Assuming an additive decomposition, we can consider our time series constructed from three components of trend, seasonal and irregular elements. So, we can describe our time series at time *t* ($y_{t}$) as:

1 $$ y_{t} = T_{t} + S_{t} + I_{t} . $$

where *T*_*t*_ is trend component at time *t* representing the long-term evolution of the concentrations. This element exists in the data when there is a persistent increase or decrease; *S*_*t*_ is the seasonal component at time *t* describing the seasonal variations in the time series that happens over a fixed, known period. The time series consists of yearly seasonality; and *I*_*t*_ is the random component at time *t* reflects local variations in the data and characterizes the remainder of the time series after removing the trends and seasonal components.

We performed the statistical analyses in R language, and we employed STL function from “seasonal” package for the decomposition. Seasonal and Trend decomposition using Loess is an adaptable, robust method for the time series decomposition, developed by Cleveland et al.^24^.

## Results and discussion

**Figure 1:**
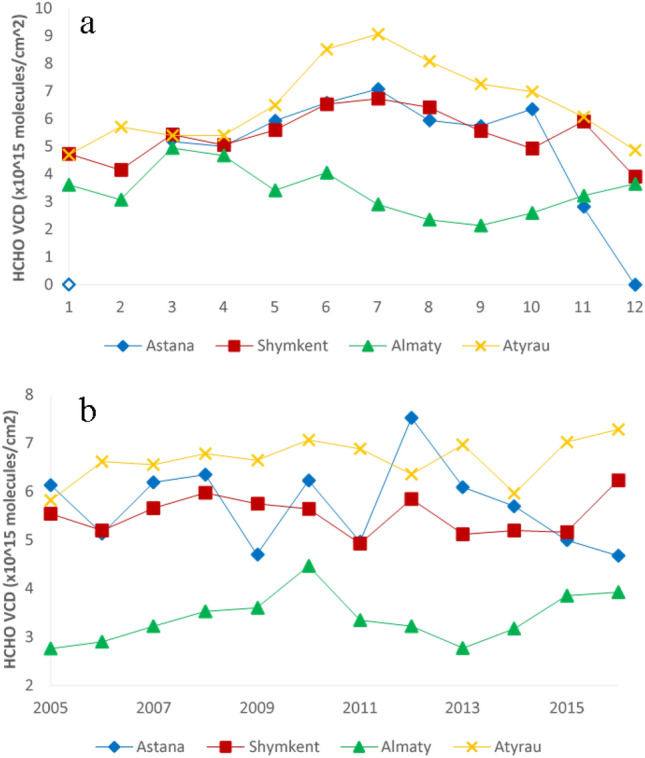
(**a**) Monthly and (**b**) annual HCHO CMD for four major cities in Kazakhstan (Nur-Sultan (Astana), Shymkent, Almaty and Atyrau).

**Figure 2:**
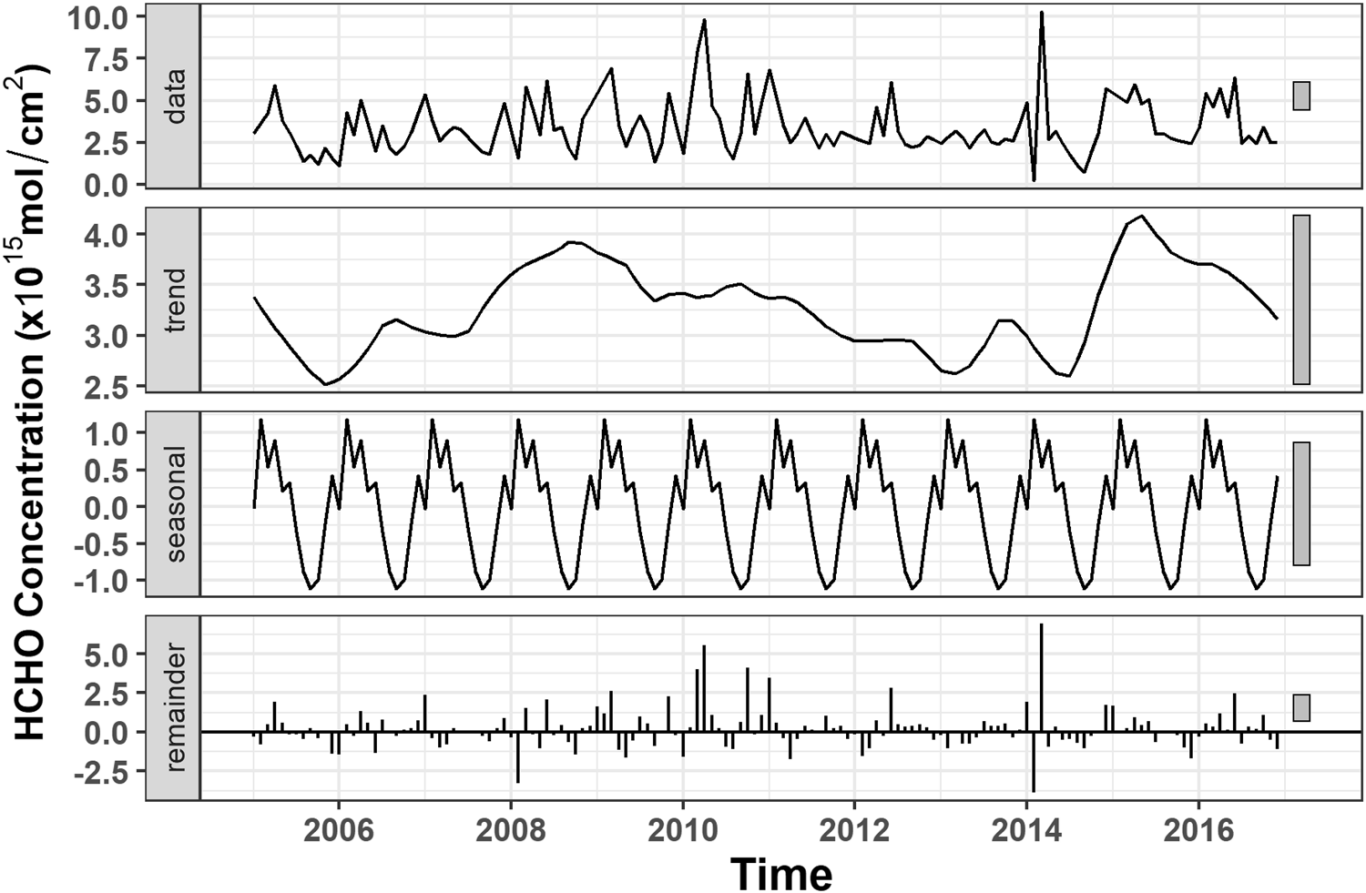
Monthly HCHO time series decomposition graph for Almaty.

The monthly mean monthly averaged for each respective month over the time period 2005–2016 and the annual variations of the satellite HCHO CMD over four cities (Nur-Sultan, Shymkent, Almaty and Atyrau) are presented in Fig. 1a,b, respectively. The maximum HCHO CMD for the three cities Nur-Sultan, Shymkent and Atyrau was observed between June and August (Fig. 1a). High photochemical activity in the summer leads to the higher production of HCHO as a major end product of atmospheric organic compound oxidation^25^. Formaldehyde increased in the summer with increased air temperature and solar intensity, and increased free radical, nitrogen oxides, ozone, and biogenic precursor concentrations. This seasonal variation of HCHO was observed for Nur-Sultan, Ekibastuz, and Atyrau. However, we observed a different trend for Almaty with higher HCHO concentrations during the spring and winter (Fig. 1a) and the lowest CMD during early Fall (September). Figure 2 presents the results of the Time Series Decomposition analyses for Almaty. It distinguishes the seasonal effects and the source effects on the monthly variations of HCHO. The seasonal effect is less influential compared to the source effect but consistently showed a maximum in winter and a minimum in summer, while the trend plot (source effect) shows both maxima and minima in summer depending on the year of the study. The observed increased CMDs in spring and winter in Almaty could be due to metrological effects including the increased solar radiation (spring) and lower boundary layer height (winter) and to a lesser extent due to sources of reactive hydrocarbons such as biomass burning (winter). While decreased HCHO in the fall could be due to increased precipitation that reduces formaldehyde via washout. The average yearly precipitation in Nur-Sultan, Ekibastuz, Atyrau and Almaty is 308 mm, 269 mm, 161 mm, and 574 mm, respectively^26^. Thus, the lower HCHO values in Almaty compared to the other cities can be attributed to the higher rates of precipitation.

Some satellite data were missing during winter for Nur-Sultan due to heavy snow covering the ground. Average monthly mean HCHO through the 12-year period ranged from 4.70 to 9.06 × 10^15^ molecules/cm^2^ for Atyrau, 2.82 to 7.07 × 10^15^ molecules/cm^2^ for Nur-Sultan, 3.90 to 6.72 × 10^15^ molecules/cm^2^ for Shymkent, and 2.13 to 4.94 × 10^15^ molecules/cm^2^ in Almaty.

Figures S9-11 present the decomposition plots for Nur-Sultan, Atyrau, and Shymkent.

**Figure 3:**
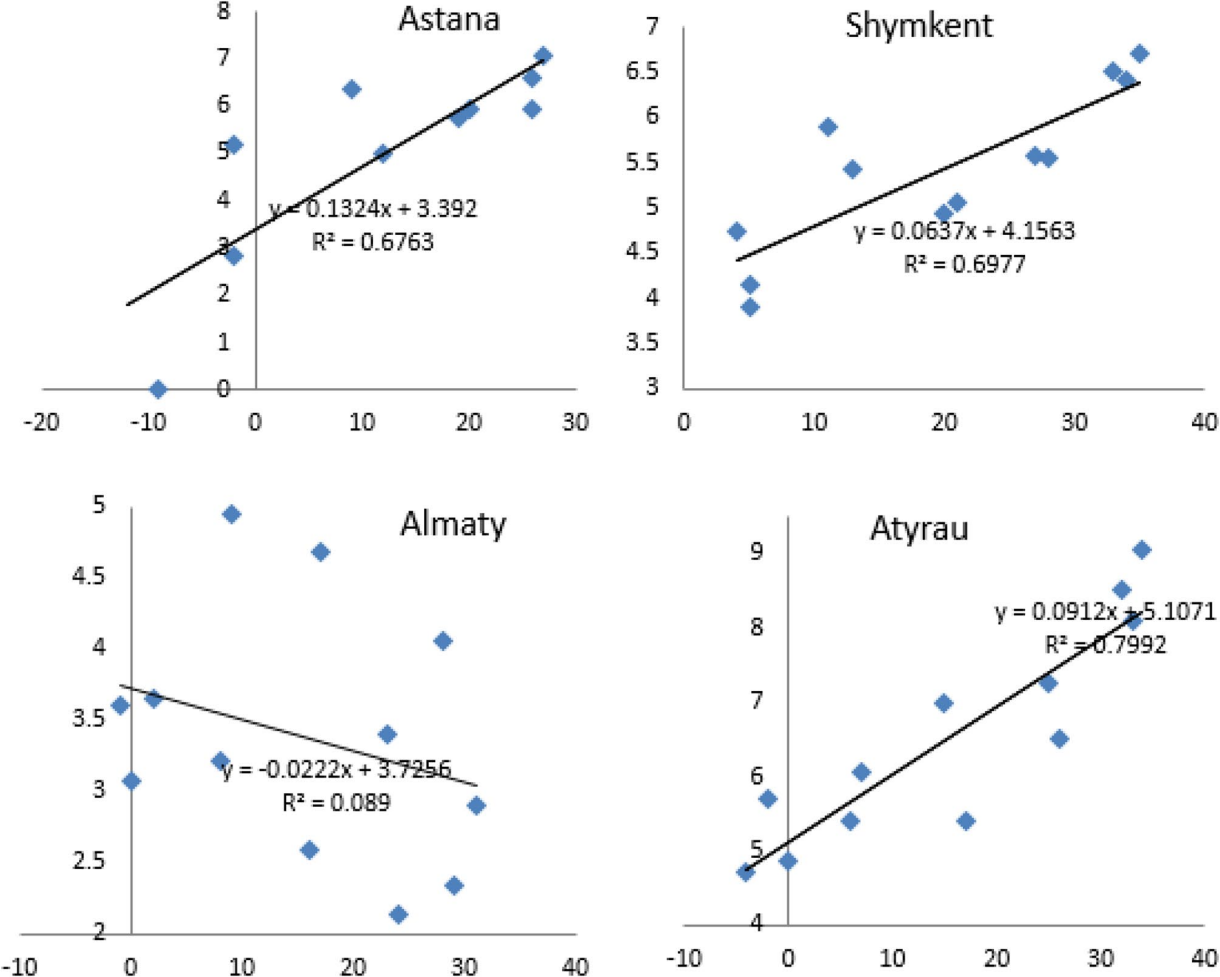
Relationship between HCHO CMD (10^15^ molecules/cm^2^ in y-axis) and average monthly temperature (°C in x-axis).

Shymkent, Almaty and Atyrau experienced slight increases between 2005 and 2016 (Fig. 1b) whereas in Nur-Sultan (blue line), the level of HCHO CMD fluctuated during the 12 years, with a peak period in 2012. The reduction of HCHO CMD in Nur-Sultan after 2012 may have resulted from the implementation of two major emissions reduction measures in 2013. Euro-4 standards were required for all new cars sold beginning this year. and an emission trading system (ETS) for greenhouse gases was implemented. Overall, during the study period, the highest average HCHO CMD was observed in Atyrau (6.66 ± 0.6 × 10^15^ molecules/cm^2^) while in Nur-Sultan and Shymkent, had very similar values (~ 5.55 ± 0.6 × 10^15^ molecules/cm^2^). The lowest HCHO CMD was estimated for Almaty (3.40 ± 0.6 × 10^15^ molecules/cm^2^). The high HCHO CMD in Atyrau can be attributed to the presence of oil and gas processing facilities that emit significant quantities of VOCs including primary HCHO. The emitted species would also serve as precursors for the formation of secondary HCHO. The relationship between monthly temperature and HCHO is depicted in Fig. 3. HCHO had very poor correlation with temperature (R^2^ = 0.09) in Almaty. However, for the other cities, Nur-Sultan, Shymkent and Atyrau, the correlation coefficients were high (0.68, 0.70, and 0.80, respectively). The high correlations between temperature and HCHO CMDs in these cities suggest that the main source of HCHO is secondary formation promoted by the higher temperatures and solar radiance. The poor correlation between temperature and HCHO CMD in Almaty may be the lack of precursor sources so that the concentrations are driven by the primary emission of HCHO by biomass burning and related combustion sources. In addition, Almaty introduced CNG vehicles in 2013 and such vehicles emit significant quantities of formaldehyde and acetaldehyde^27^.

**Figure 4:**
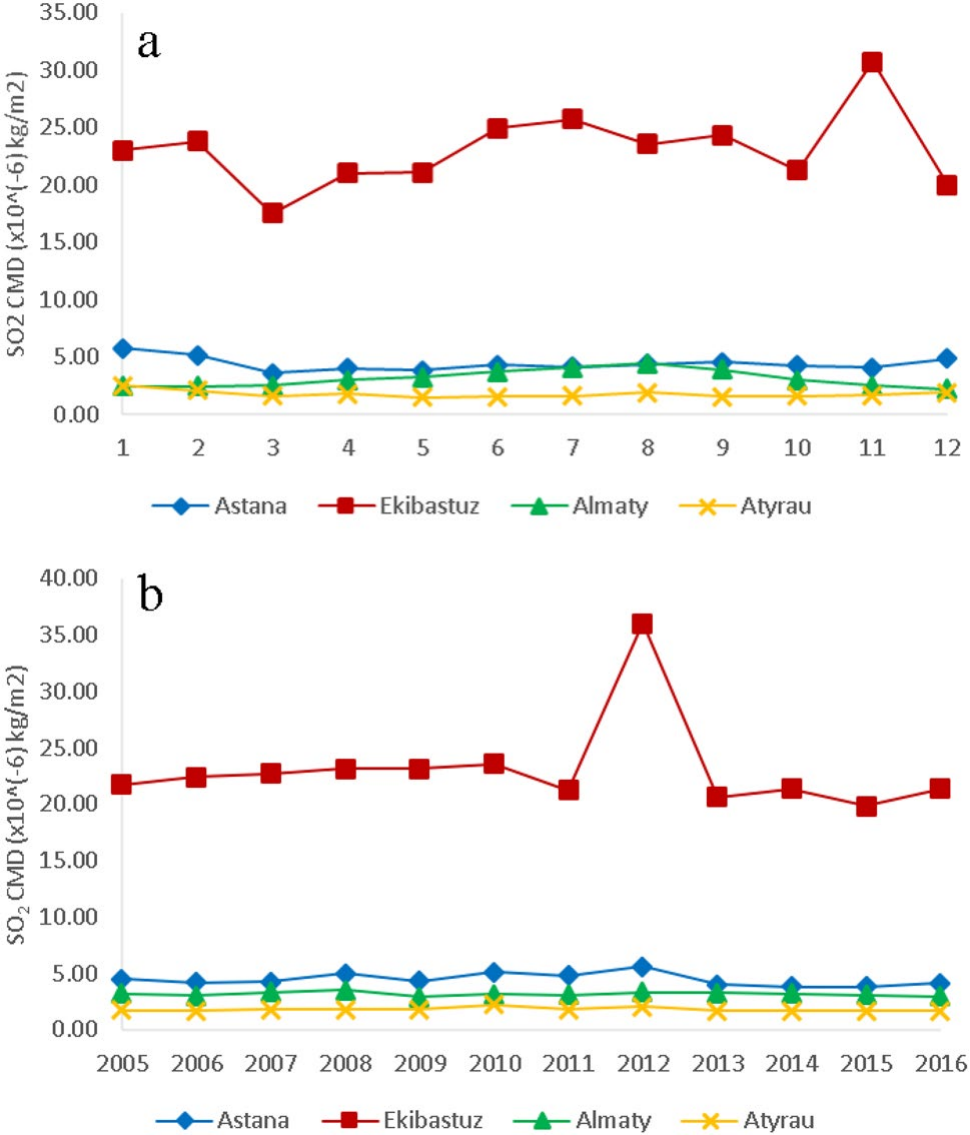
(**a**) Monthly and (**b**) annual SO_2_ CMD for four major cities in Kazakhstan (Nur-Sultan (Astana), Ekibastuz, Almaty and Atyrau).

Ekibastuz was the city that experienced the highest SO_2_ CMD likely due to two power plants, the biggest coal fired power plants in the Republic of Kazakhstan, located in the vicinity^28^. Overall, the sulfur dioxide CMD in Ekibastuz decreased slightly from 2005 to 2016 (Fig. 4a,b). However, a sharp peak in the SO_2_ CMD was observed in 2012 that can be attributed to the operation status of coal power plants (maximum capacity) in accordance with the national plan for the distribution of quotas for greenhouse gas emissions^29^. The highest energy production in the period between 2010 and 2016 was generated in 2012 with the overall generated energy in that year being more than 41.86 × 10^7^ kJ^30^. Figure S12 shows the SO_2_ CMD time series decomposition plots for Ekibastuz.

In Almaty, the main sources of SO_2_ include the burning of coal and emission from on-road vehicles. For the both cities, the trend graphs (Figures S13 and S14) show major peaks in winter. The peak in winter could be attributed to coal burning and poorer ventilation conditions (lower boundary layer heights and lower wind speeds) in Almaty. In Nur-Sultan due to the very low temperature in the wintertime, the SO_2_ CMD increases as residential coal burning increases and the dispersion conditions decline. Overall, SO_2_ CMD declined in Almaty from 2005 to 2016 with the maximum CMD of SO_2_ observed in 2008 (3.52 × 10^−6^ kg/m^2^) as shown in Figure S13 (trend plot). This peak happened after a steady increase in the CMD by approximately 10% during 2005 to 2008 followed by a sharp decrease of approximately 17% in 2009. After the financial crisis in Kazakhstan peaked in 2008, the authorities^31^ took a series of measures related to budget limitations on leading institutions such as banks, construction companies, agriculture, industrial enterprises, and others to operate at minimum capacity^32^. During the crisis, the population living in Almaty used low-quality coal due to financial restrictions and that could have resulted in the higher emissions of SO_2_ observed in 2008. Also, the combustion of cheap coal and fuel oil for heating in the winter by the residential and commercial sectors in Almaty made significant contributions to the air pollution in this city^33^.

There were relatively small variations in the SO_2_ concentrations in the other 4 cities over the study period with some decline in the more recent years. The highest peak of SO_2_ in Atyrau in the period between 2005 and 2016 was observed in 2010 (Figure S15). The observed SO_2_ CMD was 2.21 × 10^−6^ kg/m^2^ in that year. In 2010, 11.39 million tons of crude oil was processed in the three biggest oil refineries of the Republic of Kazakhstan, exceeding the planning numbers by 2% which was 381 thousand tons of crude oil according to the annual report of JSC “KazMunayGas”^34^. This over-fulfillment was achieved by assigning the extra load to the Atyrau oil refinery in 2010 (ibid), and is likely the reason for the observed peak in SO_2_ CMD in 2010.

Both formaldehyde and sulfur dioxide peaks in Nur-Sultan in 2012 could be attributed to local residential coal combustion and vehicle exhaust under poor winter dispersion condition. However, after 2012 the CMDs of these two pollutants significantly dropped.

**Figure 5:**
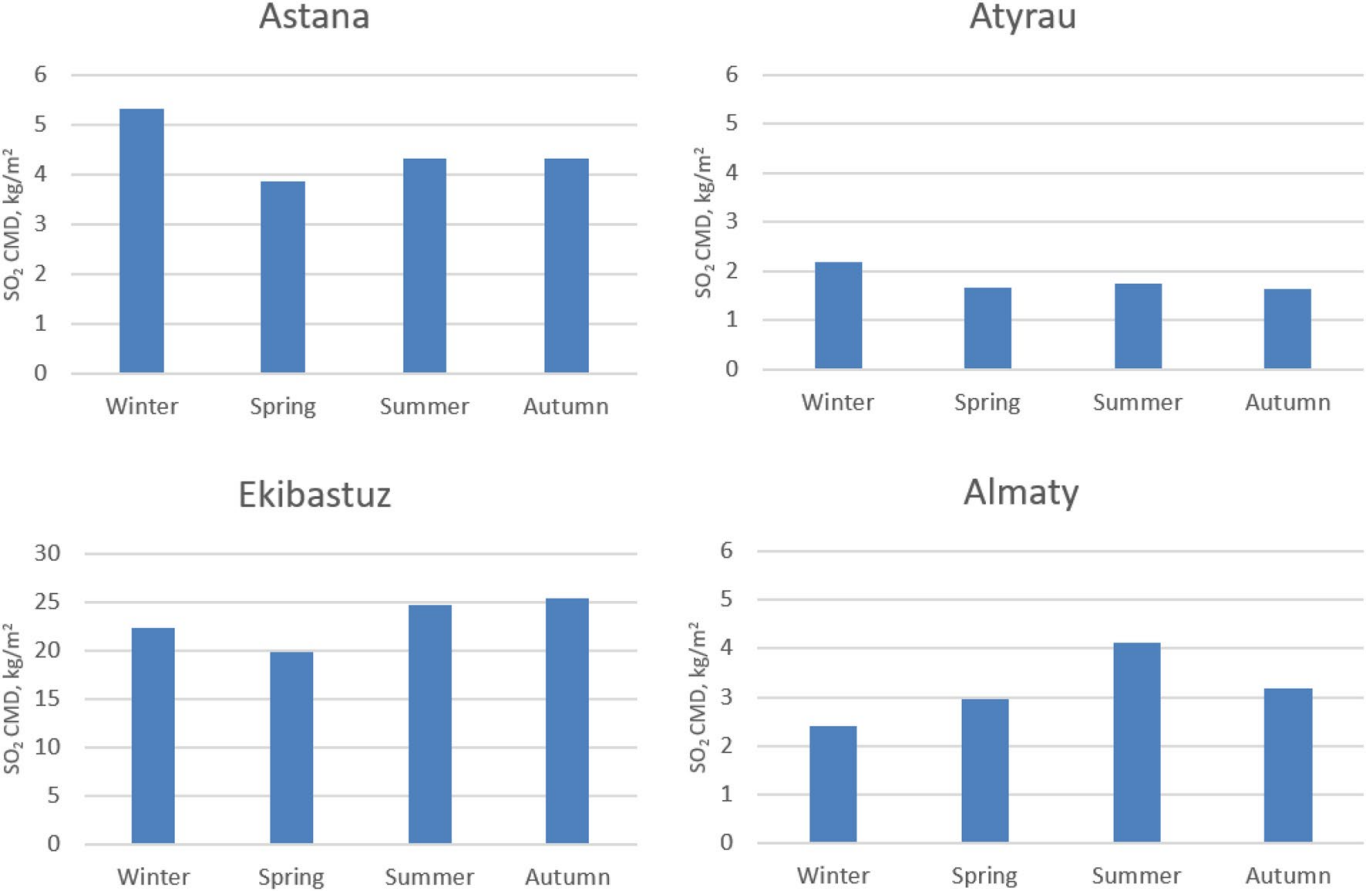
The seasonal variation of SO_2_ CMD in Nur-Sultan, Atyrau, Ekibastuz and Almaty between 2005 and 2016.

Figure 5 shows the average seasonal variations of satellite derived SO_2_ CMD between 2005 and 2016 in the four cities. High SO_2_ CMD is reported for Ekibastuz, varying between 20 and 25 kg/m^2^, with peak values in summer and fall; while in Nur-Sultan these values range between 3.9 and 5.2 kg/m^2^, in Atyrau 1.6–2.1 kg/m^2^ and in Almaty between 2.4 and 4.1 kg/m^2^. Ekibastuz is one of the largest cities near coal deposits with non-significant seasonal variations in SO_2_ CMD. Atyrau and Nur-Sultan experienced higher SO_2_ CMD in winter compared to other seasons of the year that could be attributed to the increased demand for heating energy consumption, emissions from industries and power stations during the winter period when dispersion conditions are weaker^35^. Additionally, lower concentrations of OH and H_2_O_2_ in the troposphere during the winter result in maintaining SO_2_ in winter compared to summer when OH radicals can oxidize SO_2_ to H_2_SO_4_ along with much stronger dispersion conditions with higher mixed layer heights and wind speeds^36^. Almaty shows an opposite trend with a peak in SO_2_ CMD observed in summer. The weather history shows that the major wind direction in summer in Almaty and Ekibastuz is from the North and the average wind speed is approximately 2.6 m/s and 4 m/s, respectively^37^. Ekibastuz is located north of Almaty and approximately 1,400 km distant. Transport of SO_2_ from Ekibastuz to Almaty in summer could be a factor for the increase summer SO_2_ in Almaty (Figs. 5 and S13-seasonal plot). Given the average wind speed and the direction in summer, the time required for SO_2_ to be transported from Ekibastuz to Alamty is approximately 8 days which is comparable with the atmospheric residence time of SO_2_ (7 days) in the presence of typical OH concentration^38^. This analysis suggests that SO_2_ can be transported from Ekibastuz to Almaty during summer. In winter, the wind direction in Ekibastuz is from south/west that is not in favor of SO_2_ transfer to Almaty. The higher SO_2_ concentration in summer can lead to reductions in atmospheric oxidants such as OH and therefore, secondary production of HCHO would be reduced leading in part, to the observed reductions in HCHO CMD during summer in Almaty (Fig. 1a).

## Conclusions

This study examined main SO_2_ and HCHO hot spots over Kazakhstan during the period of 2005 to 2016. Ekibastuz was found to be a hot spot for SO_2_ compared to other cities, likely due to large thermal power plants that operate in the area and provide a large portion of the electricity for Kazakhstan. High SO_2_ CMD in particular years were observed in different cities due to natural, political, and socio-economical changes including the 2007–09 financial crisis that led to the utilization of cheaper fuels such as coal and fuel oil and other factors such as abnormal number of wildfires, increased oil recycling, and electricity productions. Transferring the capital of Kazakhstan from Almaty to Nur-Sultan gave rise to the air pollution in the new capital that would be driven by urbanization and an increased population. High summer HCHO concentrations were observed over all of the studied cities except Almaty. The increases could be caused by secondary production of HCHO as a result of the photooxidation of VOCs. In Kazakhstan, the limited spatiotemporal ground monitoring means that the application of satellite data to investigate air quality is a useful and necessary method to obtain necessary air quality data.

## Supplementary information

Supplementary Information 1.
