# Supplementary material for: SO2 and HCHO over the major cities of Kazakhstan from 2005 to 2016: influence of political, economic and industrial changes
Source: Sci Rep. 2020 Jul 28;10:12635. doi: 10.1038/s41598-020-69344-w (PMC7387459; doi:10.1038/s41598-020-69344-w)
Supplement: Supplementary file 1 — Supplementary Information 1. [file 41598_2020_69344_MOESM1_ESM.docx]

**SO_2_ and HCHO over the major cities of Kazakhstan from 2005 to 2016: Influence of political, economic and industrial changes**

Zhuldyz Darynova,^1^ Mehdi Amouei Torkmahalleh,^1,2^* Talgat Abdrakhmanov,^1^ Serik Sabyrzhan,^1^ Sultan Sagynov,^1^ Philip K. Hopke,^3,4^ Jonilda Kushta^5^

^1^Chemical and Aerosol Research Team, Department of Chemical and Materials Engineering, School of Engineering and Digital Sciences, Nazarbayev University, Nur-Sultan 010000, Kazakhstan

^2^The Environment and Resource Efficiency Cluster, Nazarbayev University, Nur-Sultan 010000, Kazakhstan

^3^Department of Public Health Sciences, University of Rochester School of Medicine and Dentistry, Rochester, NY 14642 USA

^4^Center for Air Resources Engineering and Science, Clarkson University, Potsdam, NY 13699 USA

^5^The Cyprus Institute, Energy Environment and Water Research Center, 2121, Nicosia, Cyprus

Corresponding author:

^*^Mehdi Amouei Torkmahalleh (e-mail: mehdi.torkmahalleh@nu.edu.kz phone: 0077172702647)

Almaty (43.22^o^N, 76.85^o^E) is the biggest city of Kazakhstan with a population of almost 2 million people ("Statistics", 2018). The location of Almaty is unique since it is situated in the valley of the Trans-Ili Ala-Tau Mountains (Figure S1). The specific location of Almaty limits the movement of the horizontal wind which might cause the accumulation of the emissions. According to Carlsen et al. (2013), in Almaty, during the daytime, polluted air in the north moves to the south due to common direction of the wind that blows from the north to the south. The reverse pattern is seen during the nighttime.

Nur-Sultan (51.16^o^N, 71.47^o^E) is the capital city of Kazakhstan which is located in the North part of Kazakhstan. After becoming the capital city of Kazakhstan, the population started to increase rapidly. As can be seen in Figure S3, the agglomeration of Nur-Sultan from 2000 to 2018 increased by 170%. This caused a dramatic increase in the construction sector, road and railway transportation and the number of vehicles in the city and hence, negative impacts on the air quality. The main source of the sulfur dioxide and VOCs comes from the power plants that operate by burning the coal.  The location of the power plants in Nur-Sultan can be seen in Figure S4.

In December 1997, Kazakhstan’s capital city changed from Almaty to Nur-Sultan (51.16°N, 71.47°E). As a result industrial outputs and the gross regional products have increased by 11 and 90 times, respectively. While the trend of migration to Almaty did not decrease from 2005 to 2016, the population migrating to the new capital, Nur-Sultan, increased. This city development as a result of the political change in the country resulted in increases in the air pollution level in Nur-Sultan. Figure S3 shows the population increase by 65% from 2005 to 2016 in Nur-Sultan (Darynova et al. 2018). This shift in capital, construction, and transport by roads and railways increased dramatically, introducing increased sources of dust and air pollutions. To offset air pollution, the government organized projects such as the construction of a large (190 ha) forest park. In addition, the area of landscaping in Nur-Sultan increased from 67.9 ha in 1997 to 1061.5 ha in 2009. Figure S4 shows the current map of Nur-Sultan.

Ekibastuz (51.73^o^N, 75.32^o^E) is the city that is located in the region of Pavlodar in northeastern Kazakhstan (Figure S5). The population of Ekibastuz was increased by 8% from 2003 to 2018 (Figure S6). Ekibastuz has one of the most massive coal deposits among cities of Kazakhstan. The coal in the Ekibastuz is mined by applying the open cut mining method and extracted coal is used to fuel two largest coal power plants in Kazakhstan which contribute to the 15% of all generated electricity in the country (Qalmykov and Malikova, 2017). In the period between 2012 and 2015, both coal plants that situated near the Ekibastuz city generated approximately 79 billion kilowatt hours of electricity (Qalmykov and Malikova, 2017). Therefore, a significant amount of SO_2_ and VOCs emits to the atmosphere due to burning the high-ash coals to generate electricity. Brown coal that is burned in coal power plants in Ekibastuz consist of 30% toxic minerals with a long lifetime.

Atyrau (47.11^o^N, 51.52^o^E) is the unofficial oil capital of Kazakhstan which is located near the Caspian Sea (Figure S7). According to Figure S8, the population of Atyrau increased by almost 16% in the period between 2010 and 2018. Atyrau has two massive oil fields such as Tengiz onshore oil field and Kashagan offshore oil field. Also, one of the largest oil refinery plants of Kazakhstan exists in Atyrau. The main source of the SO_2_ emission in the atmosphere of Atyrau comes from Atyrau oil refinery which also emits VOCs (Tasboulatova 2013).


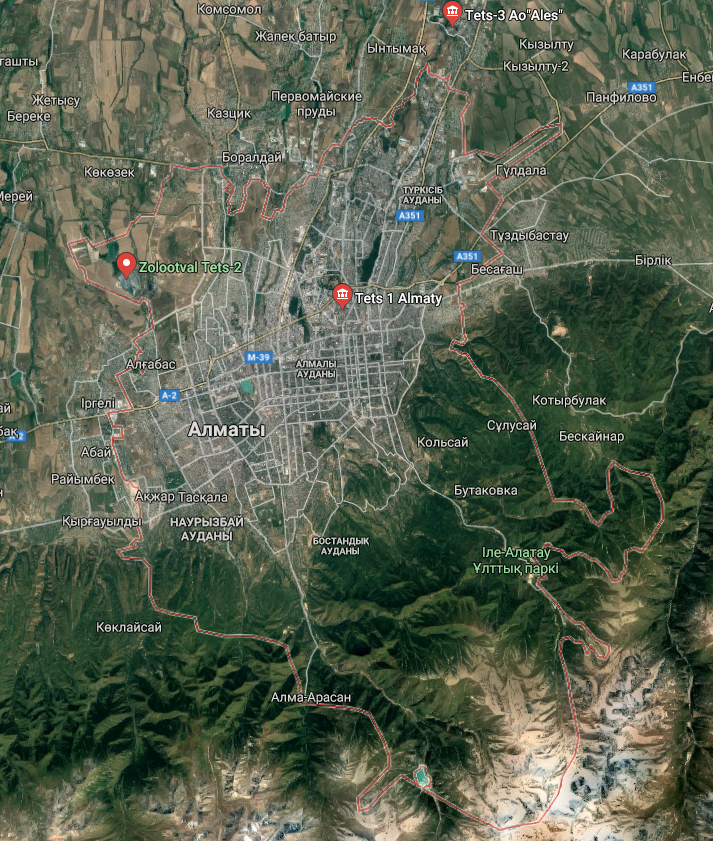


Figure S1. The location of Almaty with locations of power plants obtained from the Google map (Google Map, n.d) and Figure S2. Almaty population from 2000 to 2018 ("Statistics" 2018)


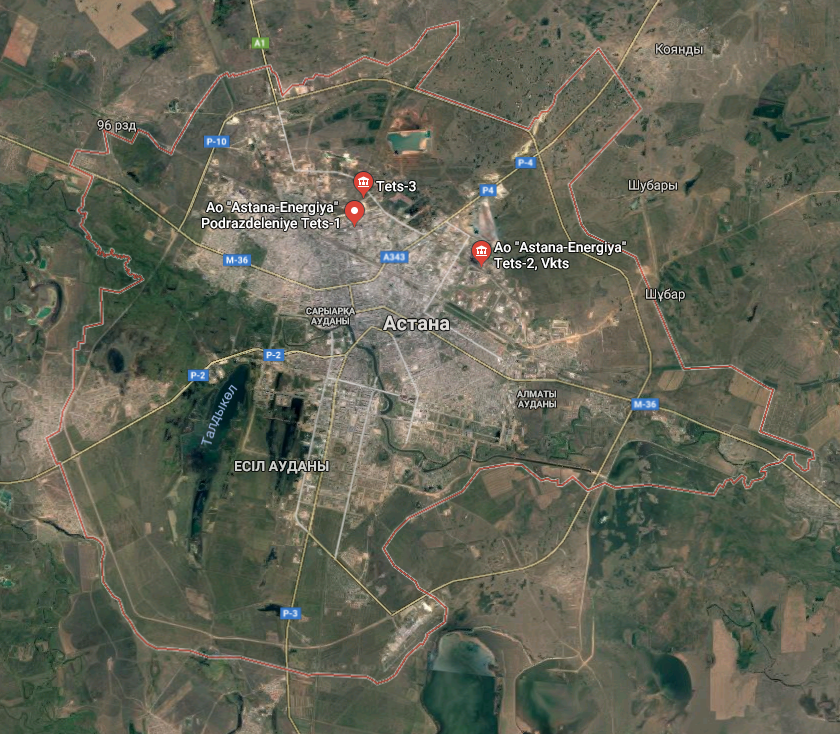


Figure S3. Nur-Sultan population from 2000 to 2018 ("Statistics" 2018) and

Figure S4. The location of Nur-Sultan with locations of power plants obtained from the Google map (Google Map, n.d)


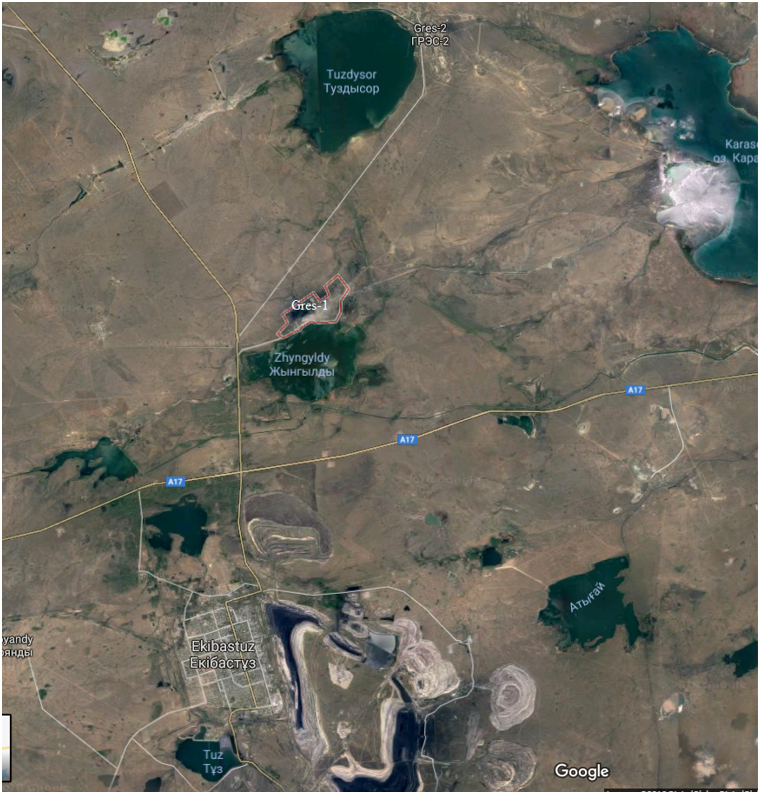


Figure S5. Location of Ekibastuz with locations of power plants obtained from Google map (Google Map, n.d) and Figure S6. Ekibastuz population from 2003 to 2018 ("Population Statistics" 2018)


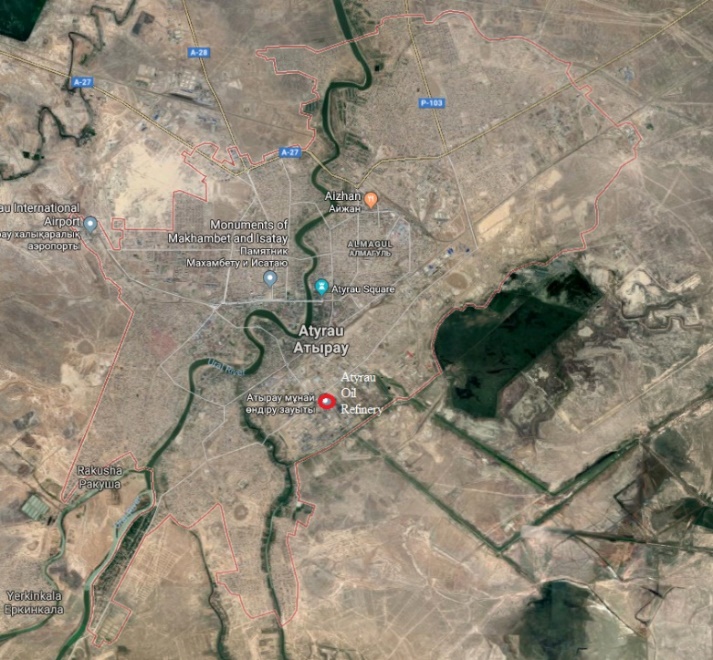


Figure S7. Location of Atyrau with location of oil refinery obtained from Google map (Google Map, n.d) and Figure S8. Atyrau population from 2000 to 2018 (“Statistics” 2018)


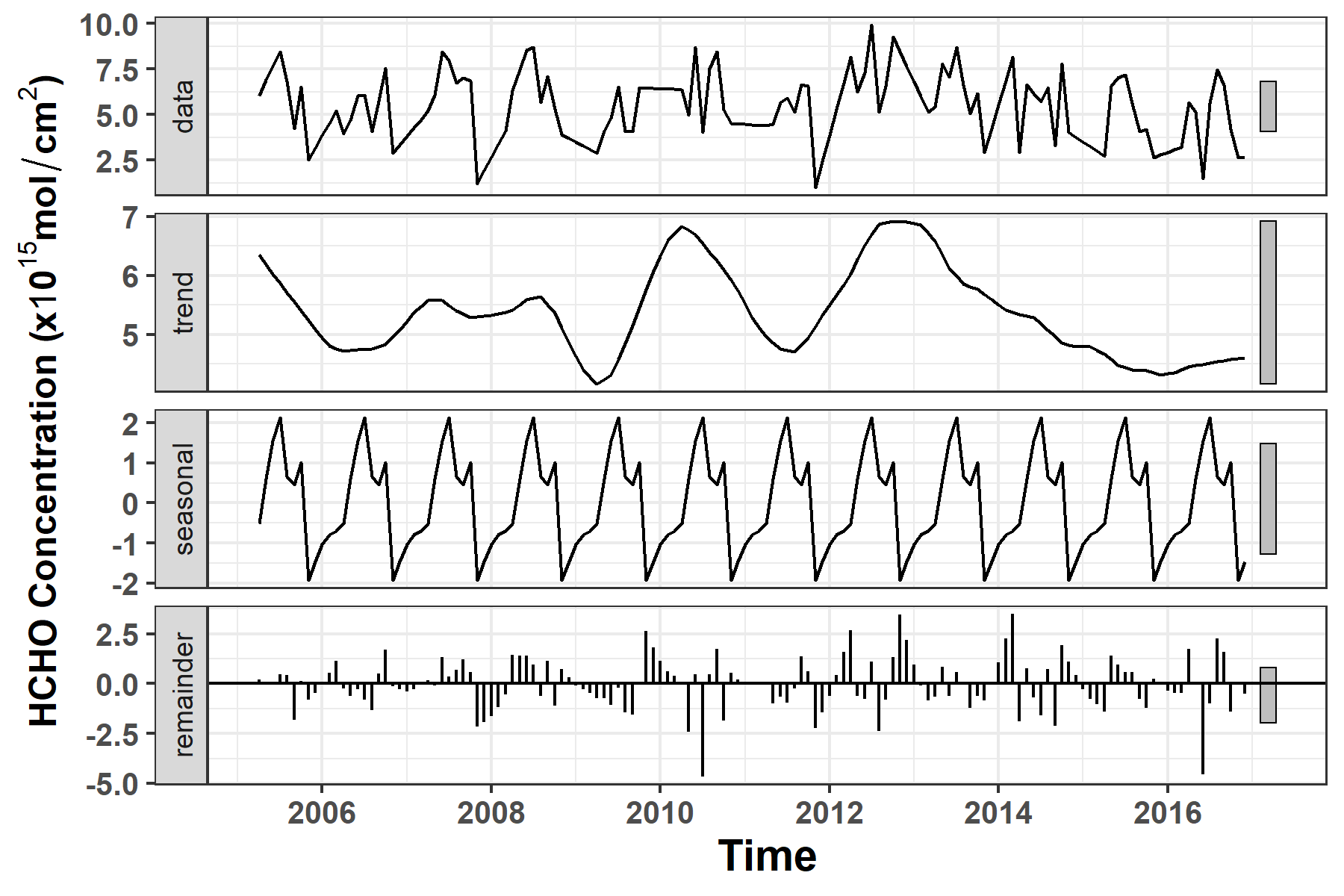


Figure S9. Monthly HCHO Time Series Decomposition Plot for Nur-Sultan


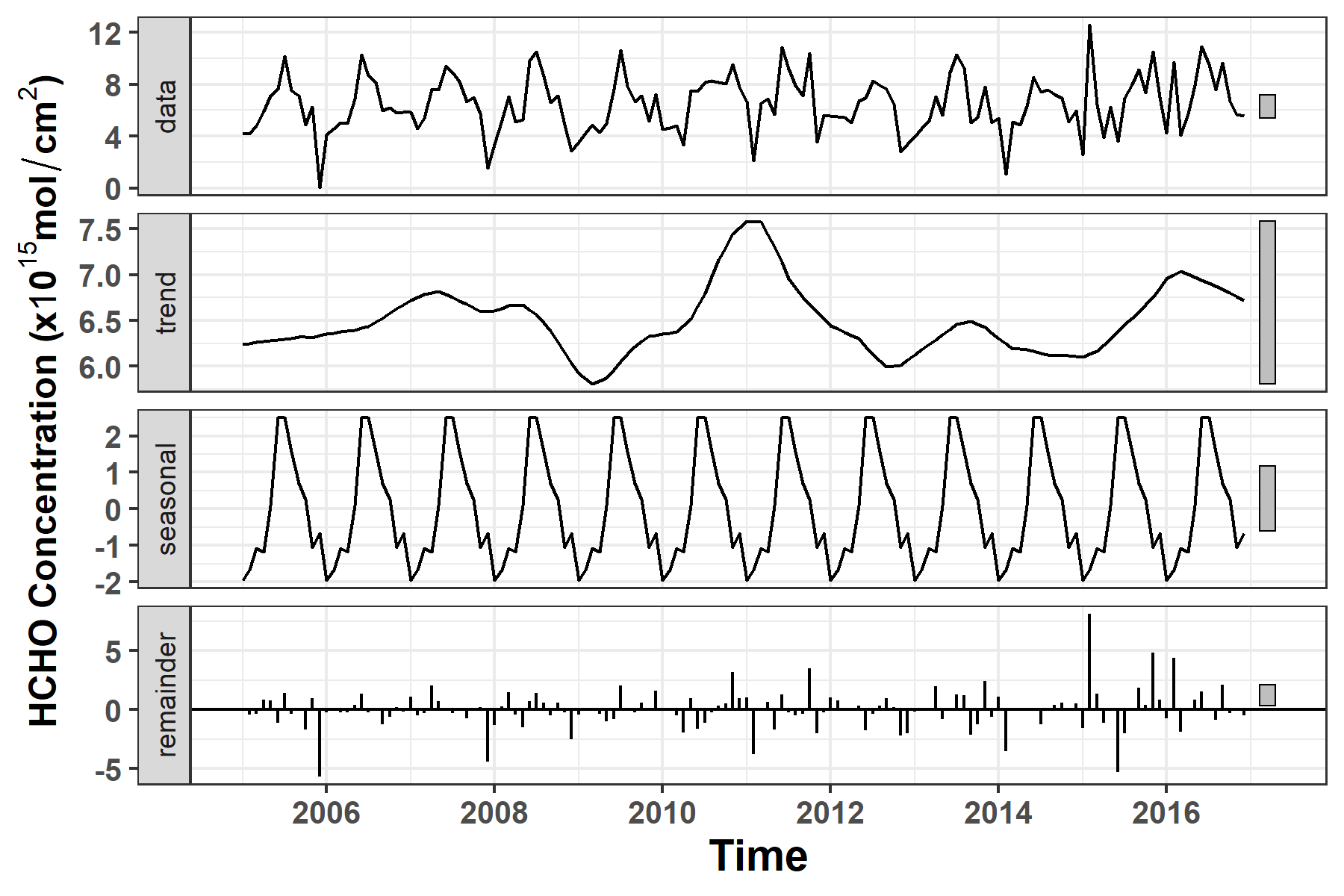


Figure S10. Monthly HCHO Time Series Decomposition Plot for Atyrau


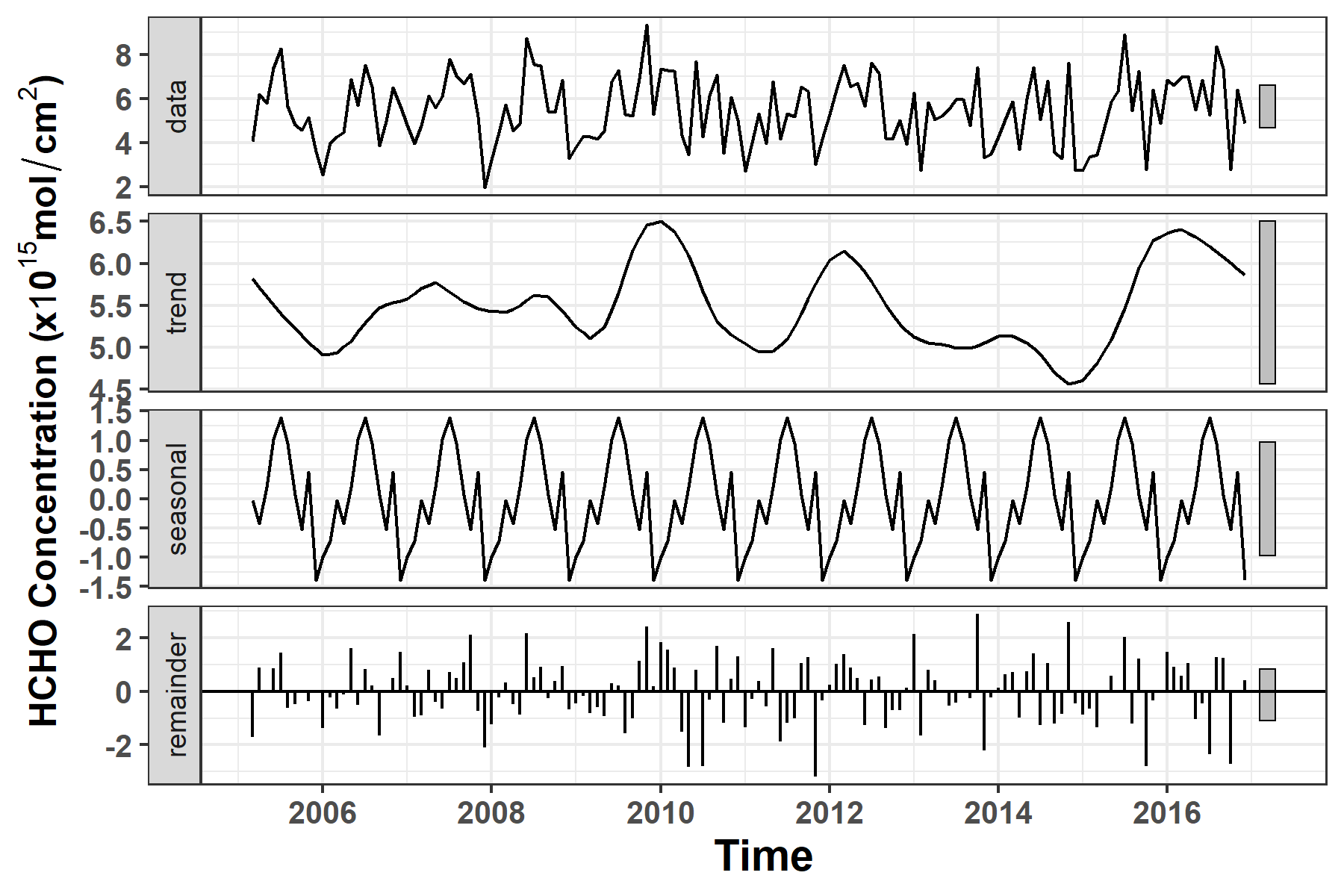


Figure S11. Monthly HCHO Time Series Decomposition Plot for Shymkent


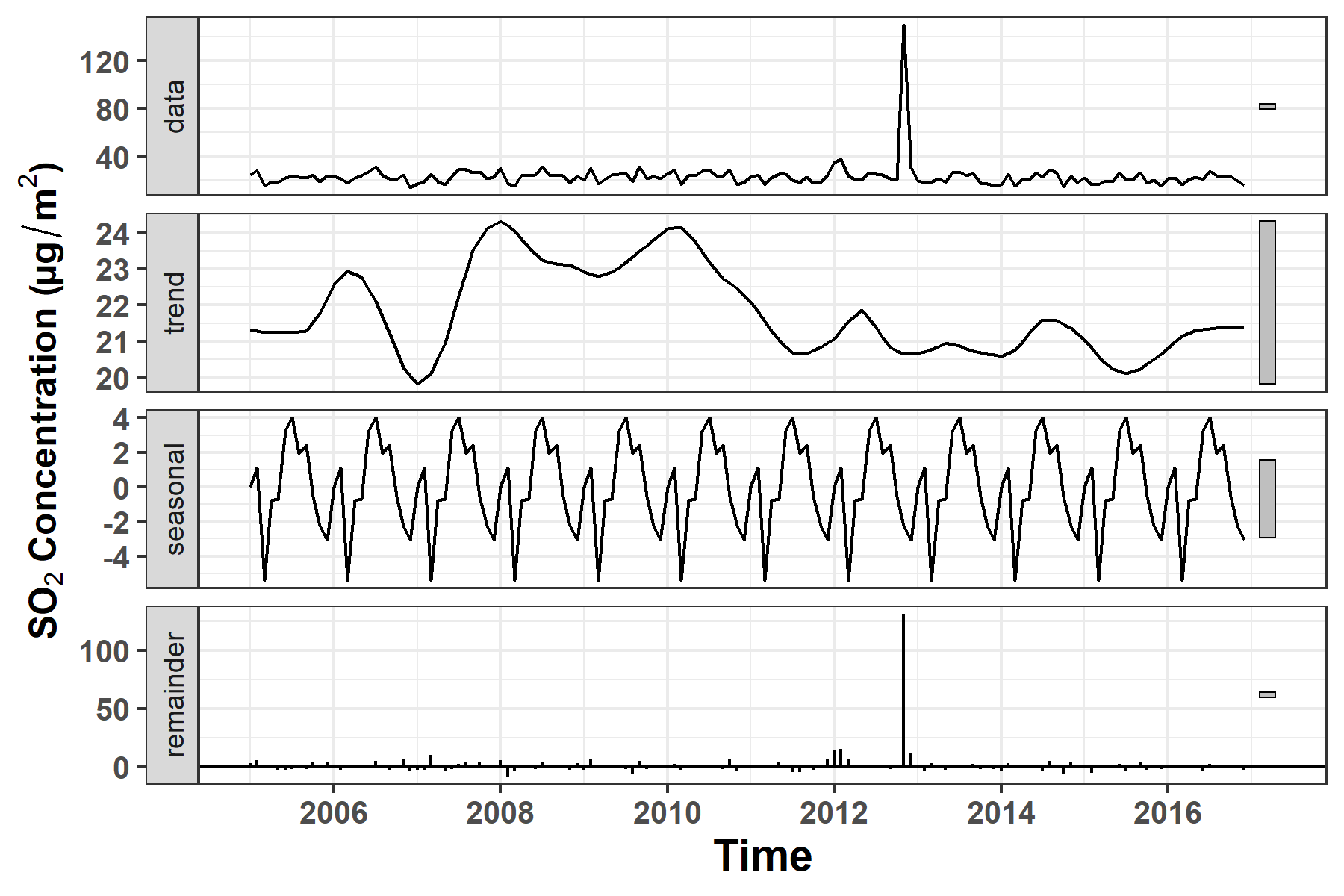


Figure S12. Monthly SO_2_ Time Series Decomposition graph for Ekibastuz


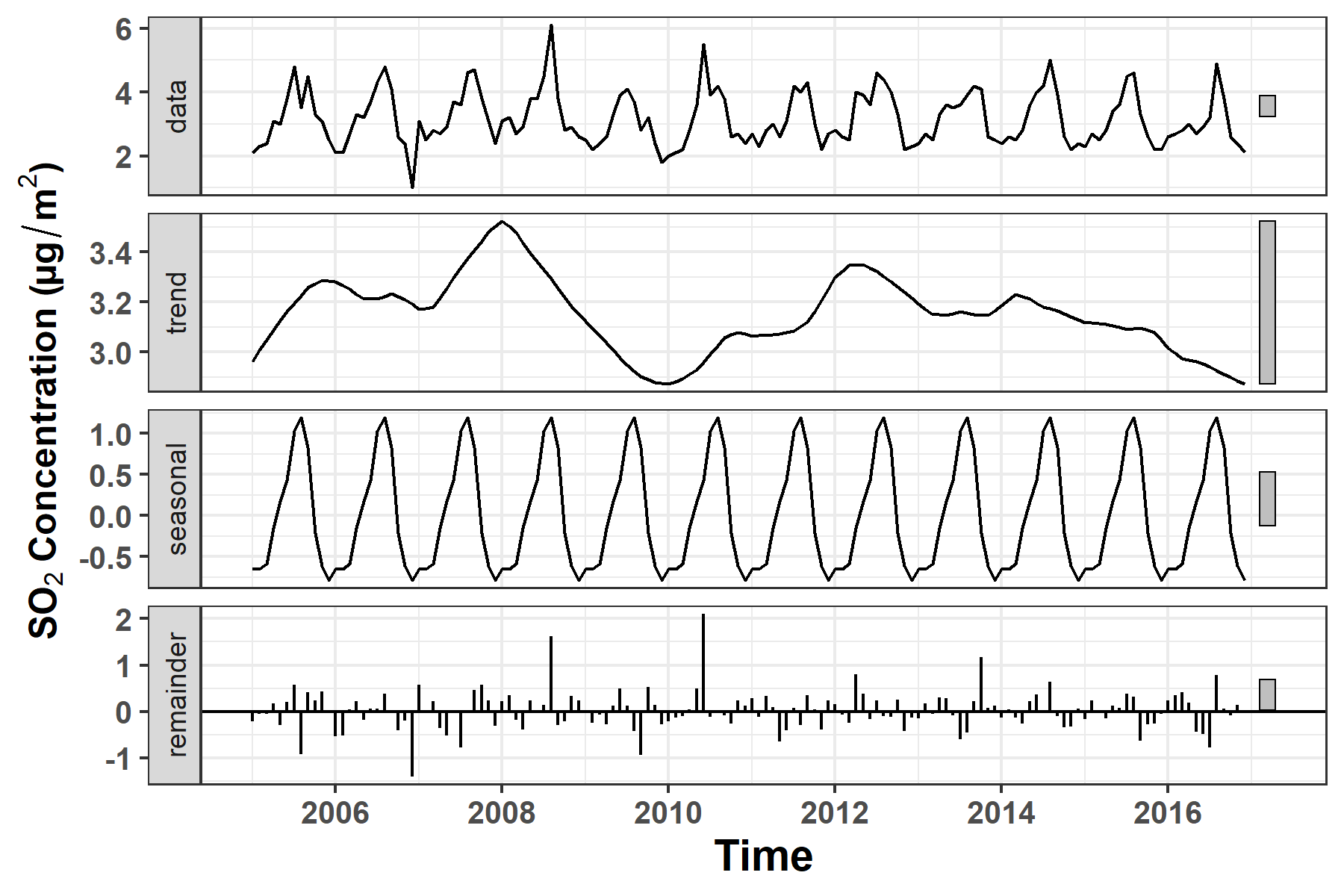


Figure S13. Monthly SO_2_ Time Series Decomposition graph for Almaty


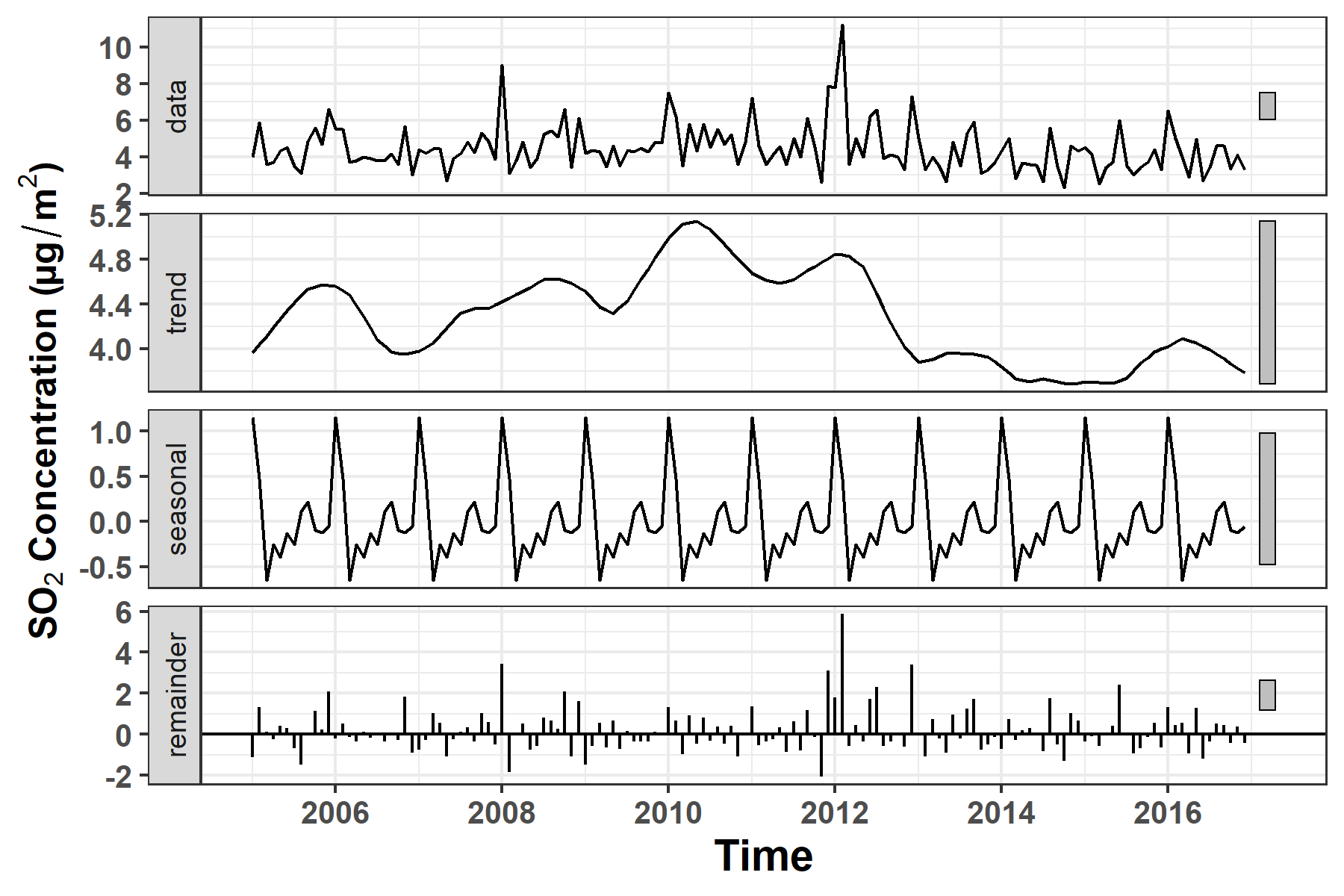


Figure S14. Monthly SO_2_ Time Series Decomposition graph for Nur-Sultan


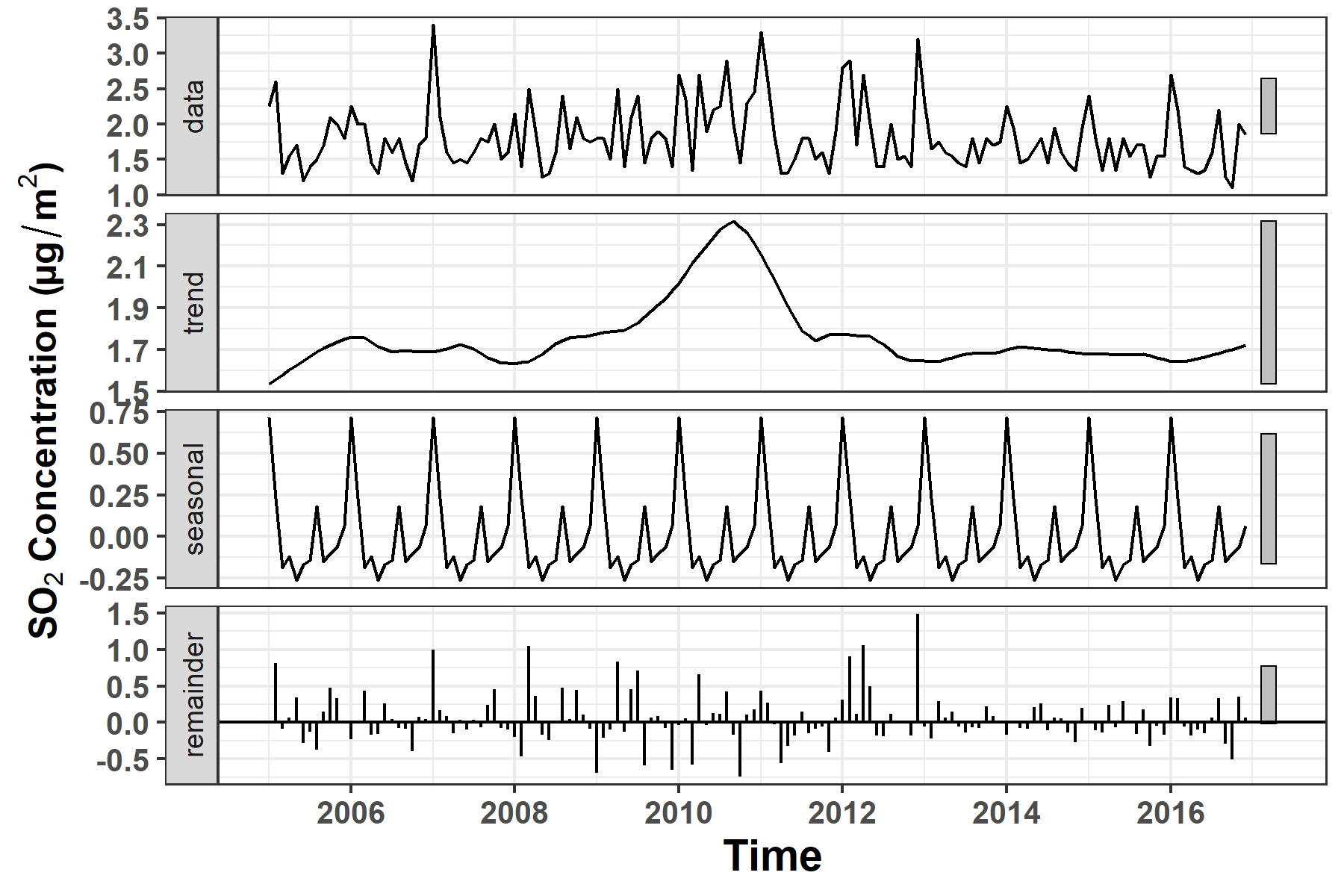


Figure S15. Monthly SO_2_ Time Series Decomposition graph for Atyrau
